# Chicken vaccination reduces colonization and dissemination of *Salmonella* serovar Enteritidis with decreased susceptibility to ciprofloxacin

**DOI:** 10.1038/s41541-026-01414-y

**Published:** 2026-03-09

**Authors:** Bradley L. Bearson, Samuel J. Whelan, Maya P. N. Encinosa, Durga P. Neupane, David J. Bradshaw, Melissa S. Monson, Christopher L. Anderson, Shawn M. D. Bearson

**Affiliations:** 1 Agroecosystems Management Research Unit, USDA, ARS, National Laboratory for Agriculture and the Environment, Ames, IA USA; 2 Food Safety and Enteric Pathogens Research Unit, USDA, ARS, National Animal Disease Center, Ames, IA USA; 3 Agricultural Research Service Participation Program, Oak Ridge Institute for Science and Education, Oak Ridge, TN USA

**Keywords:** Diseases, Immunology, Microbiology

## Abstract

*Salmonella enterica* serovar Enteritidis (*S*. Enteritidis) is one of most common *Salmonella* serovars associated with human illness in the U.S. and worldwide. Surveillance from the U.S. National Antimicrobial Resistance Monitoring System indicates an increase in both chicken and human isolates of *S*. Enteritidis with decreased susceptibility to ciprofloxacin (DSC), a critical antibiotic prescribed for complicated human salmonellosis infections. *S*. Enteritidis reduction in chickens is a priority of poultry producers and public health agencies to improve food safety. In the current study, efficacy assessment of a live *Salmonella* vaccine (BBS 1134) revealed significant reduction of cecal and splenic colonization, and prevention of dissemination to the bone marrow by DSC *S*. Enteritidis in broiler chickens. Microbiome analysis indicated the cecal microbiota of vaccinated chickens is distinct compared to mock-vaccinated birds. The IDEXX SE Ab X2 Test did not detect antibodies to *S*. Enteritidis in vaccinated chicken serum, thereby permitting differentiation of infected from vaccinated animals (DIVA). Altogether, the *Salmonella* vaccine is a DIVA vaccine, afforded cross-protection, and significantly reduced intestinal colonization and dissemination to the spleen and bone marrow by DSC *S*. Enteritidis in chickens, thereby offering a prospective intervention for animal production to reduce food product contamination and improve food safety.

## Introduction

*Salmonella enterica* is a major cause of human foodborne illness, estimated at 80.3 million cases globally each year^1^. The commensal nature of *Salmonella* results in food animals serving as sub-clinical reservoirs, complicating detection and mitigation on the farm. *Salmonella enterica* subspecies *enterica* serovar Enteritidis (*S*. Enteritidis) is one of the most common serovars in the United States and worldwide, and chicken products are frequently implicated in human foodborne illness with *S*. Enteritidis^2–5^. A recent investigation demonstrates that *S*. Enteritidis is one of the top five *Salmonella* serovars isolated in U.S. poultry processing plants^6^. Current surveillance from the U.S. National Antimicrobial Resistance Monitoring System (NARMS) indicates an upward trend of decreased susceptibility to ciprofloxacin (DSC) in *S*. Enteritidis isolated from chicken products and humans^7^. In 2016, 100% (*n* = 499) of *S*. Enteritidis strains isolated from chicken product and cecal samples from federally inspected U.S. slaughter establishments were susceptible to ciprofloxacin; in 2024, 64% (229/358) of isolates had DSC^7^. Ciprofloxacin is one of a limited number of antibiotics used to treat severe *Salmonella* infections in humans^8^, and reduced efficacy against *S*. Enteritidis could result in increased treatment failure for this common serovar.

The prevalence of *S*. Enteritidis and emergence of DSC *S*. Enteritidis in broiler chickens support the need for intervention methods to limit risks of this *Salmonella* of concern^6,7^. Effective interventions at the pre-harvest stage of production reduce contamination in the food supply chain, from the farm environment to the processing plant. Our team designed, tested, and reported on a live attenuated vaccine strain (BBS 866) with a *S*. Typhimurium backbone (serogroup B) that is cross-protective against multiple *Salmonella* serovars in different serogroups in both turkeys and swine^9–11^. Furthermore, antibodies induced in the BBS 866-vaccinated swine do not interfere with monitoring for natural *Salmonella* infections, indicating that the vaccine strain allows the differentiation of infected from vaccinated animals (DIVA)^10,11^. In the current study, DIVA status of the BBS 1134 *Salmonella* vaccine strain (a derivative of BBS 866) was confirmed in broiler chickens for *S*. Enteritidis. Furthermore, BBS 1134 vaccination reduced cecal and splenic colonization, and prevented dissemination of DSC *S*. Enteritidis (serogroup D) to the bone marrow in chickens, illustrating cross-protection without surveillance interference, which are desirable vaccination traits for poultry producers.

## Results and discussion

Previous research investigations in swine and turkeys demonstrated that a live attenuated *S*. Typhimurium (serogroup B) vaccine strain BBS 866 is cross-protective against multiple *Salmonella* serovars from different serogroups^9–11^. The BBS 1134 vaccine strain is a kanamycin-sensitive derivative of BBS 866^12^; deletion of the *neo* gene, conferring kanamycin resistance, was performed as previously described^13^. The goal of the current study was to determine if the serogroup B *S*. Typhimurium vaccine strain BBS 1134 cross-protects broiler chickens against DSC *S*. Enteritidis from serogroup D. *S*. Enteritidis strain SX514 (FSIS12211648) was isolated from comminuted turkey in 2022 by the USDA Food Safety and Inspection Service (FSIS). Whole genome sequencing identified SX514 as having DSC due to a missense mutation in the *gyrA* gene conferring D87Y in the encoded DNA gyrase. DSC *S*. Enteritidis strain SX514 is classified by the U.S. Centers for Disease Control and Prevention (CDC) as a reoccurring, emerging, or persisting (REP) strain (REPJEG02) and is a member of SNP cluster PDS000065758 in the NCBI Pathogen Detection Project, which also contains *S*. Enteritidis isolates from human clinical, chicken thighs, and raw intact chicken samples, suggesting that poultry-associated strains are a potential source for human clinical disease.

### Vaccination reduces DSC *S*. Enteritidis colonization in chickens

**Fig. 1:**
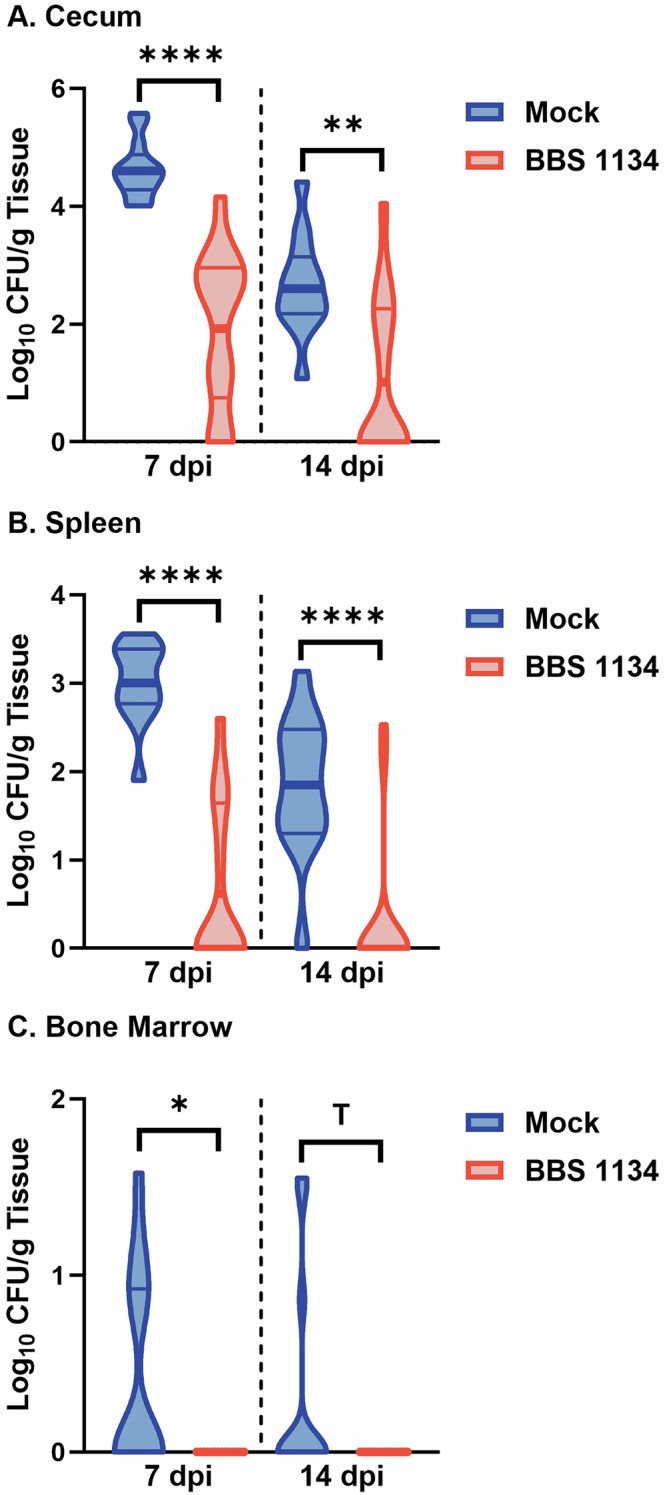
Reduction of DSC *Salmonella enterica* serovar Enteritidis colonization of the cecum, spleen and bone marrow in BBS 1134-vaccinated chickens. Chicks were administered the BBS 1134 vaccine or PBS (mock-vaccination) at 1-day of age via aerosol and booster vaccinated at 2-weeks of age via communal water administration. At 5-weeks of age, chickens were inoculated with DSC *S*. Enteritidis strain SX514. At 7- or 14-days post-inoculation (dpi) tissues (cecum (**A**), spleen (**B**), and bone marrow (**C**)) were harvested for evaluation of *Salmonella* colonization. The colony forming units (CFU) of *Salmonella* per gram (g) of tissue were determined and the results following Log_10_ transformation are presented as a frequency distribution of the data. The thick line represents the mean and the thin lines indicate the quartiles. Statistically significant differences between groups are represented by *P ≤ 0.05, **P ≤ 0.01, ****P ≤ 0.0001 and ^T^*P* ≤ 0.1.

**Fig. 2:**
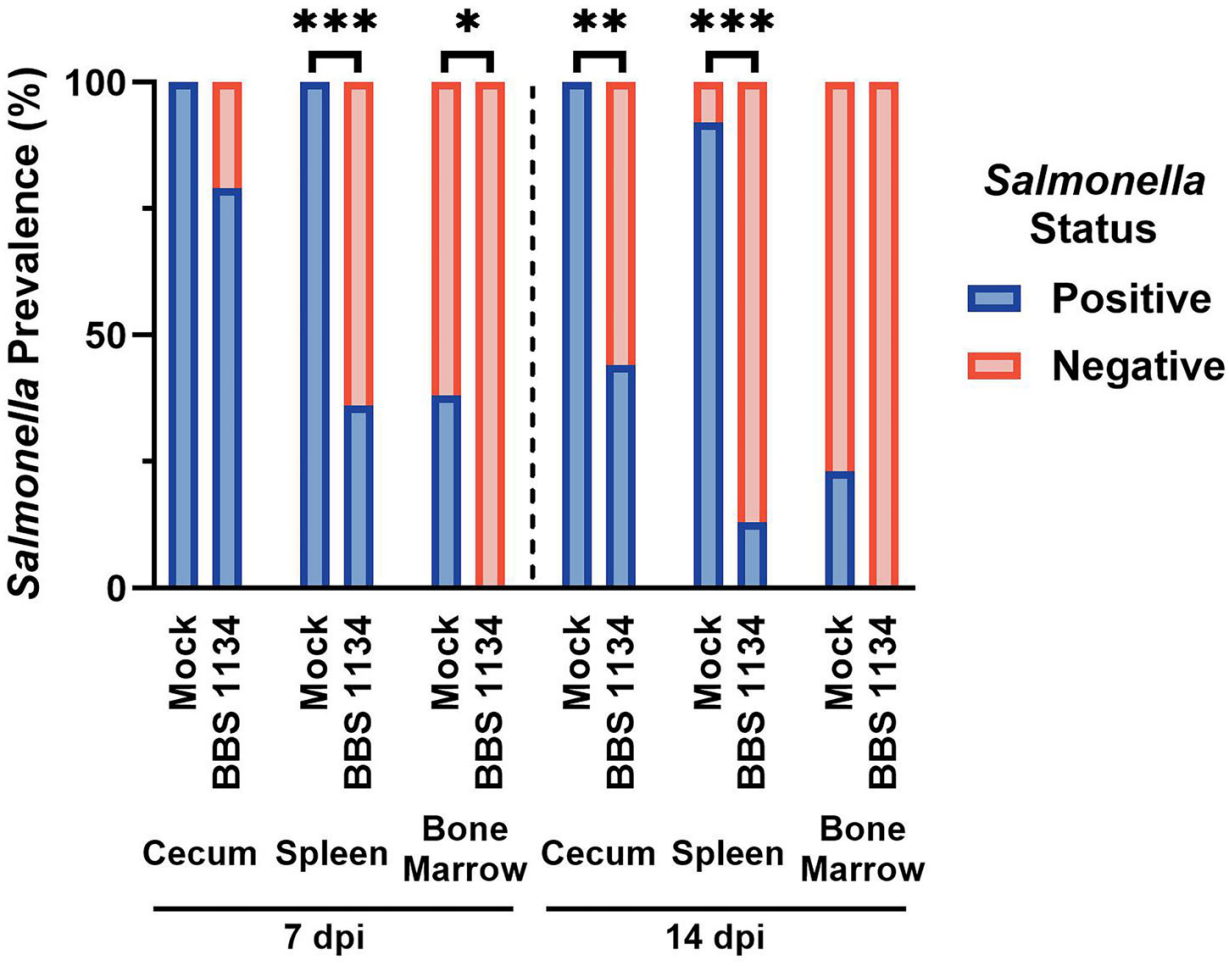
Reduced prevalence of DSC *Salmonella enterica* serovar Enteritidis in tissues of BBS 1134-vaccinated chickens. The relative percentage of DSC *S*. Enteritidis strain SX514 in positive and negative tissue samples of cecum, spleen, and bone marrow for mock-vaccinated and BBS 1134-vaccinated chickens at 7 and 14 dpi. Qualitative data was obtained based on procedures and analysis performed as described in Fig. 1 legend. Statistically significant differences between groups are represented by *P ≤ 0.05, **P ≤ 0.01, and ***P ≤ 0.001.

Chicks were vaccinated or mock-vaccinated at 1 day of age and booster vaccinated at 2-weeks of age with vaccine strain BBS 1134 or PBS, respectively. Prevalence of the vaccine strain BBS 1134 in cecal contents at 5-weeks of age was 2/12 (17%) chickens. All chickens were inoculated via oral gavage with DSC *S*. Enteritidis strain SX514 at 5-weeks of age, and *Salmonella* load was assessed at 7- and 14-days post-inoculation (dpi) in cecum, spleen, and bone marrow tissues. Quantitative analysis revealed that vaccination significantly reduced colonization of DSC *S*. Enteritidis SX514 in the cecum by >2.2Log_10_ CFU/g tissue at 7-dpi (*p* < 0.0001) and >1.5Log_10_ at 14-dpi (*p* = 0.0019) (Fig. 1A). Systemic dissemination of SX514 to the spleen was significantly reduced ~2.4 and ~1.5Log_10_ CFU/g tissue at 7- and 14-dpi, respectively, (both *p* < 0.0001) in vaccinated chickens compared to mock-vaccinated chickens (Fig. 1B). Dissemination of SX514 to the bone marrow was detected in mock-vaccinated chickens at both 7- and 14-dpi; however, vaccination with BBS 1134 prevented dissemination of SX514 to the bone marrow at both 7- and 14-dpi (Fig. 1C). At 7 dpi, the prevalence (i.e., presence/absence) of SX514 in the bone marrow was significantly (*p* = 0.0159) lower in the vaccinated chickens (0/14) compared to mock-vaccinated chickens (5/13), as was also observed for the spleen (*p* = 0.0006) in the vaccinated (5/14) versus mock-vaccinated (13/13) groups (Fig. 2). At 14 dpi, prevalence of SX514 in the spleen was significantly (*p* < 0.0001) lower in the vaccinated chickens (2/16) compared to the mock-vaccinated group (12/13); significantly (*p* = 0.0012) lower prevalence was observed in the cecum of vaccinated (7/16) compared to mock-vaccinated (13/13) chickens, and a trend (*p* = 0.0783) towards a reduction of SX514 dissemination to the bone marrow in vaccinated (0/16) compared to mock-vaccinated (3/13) chickens was noted (Fig. 2). Together, these data indicate that vaccination with *S*. Typhimurium vaccine strain BBS 1134 significantly reduced cecal and splenic colonization of chickens by DSC *S*. Enteritidis strain SX514 and prevented dissemination to the bone marrow.

Current *Salmonella* vaccination procedures in poultry production use spray vaccination at 1–2 days of age and a water-delivered, booster vaccination several weeks later; this practice allows for efficient vaccination of thousands of birds with minimal handing. However, research investigations frequently vaccinate via oral gavage, a method of delivery that ensures each bird receives a consistent vaccine dose for stimulation of the immune system. The *Salmonella* vaccination protocol employed in the current study simulated vaccine administration performed by the poultry industry, which may have introduced some variability in vaccination efficacy. Inoculation of chickens with DSC *S*. Enteritidis via oral gavage ensured a consistent challenge dose for each bird regardless of vaccination status for confident comparison of the two treatment groups.

*S*. Enteritidis is known to disseminate to internal organs (liver, spleen, ovary, oviduct) in chickens^14^. Alali et al. suggest that *Salmonella* contamination of mechanically separated chicken (MSC) products, made by grinding bone-in chicken parts, may be predicted by the presence of *Salmonella* in composite spleen samples harvested at slaughter^15^. Another potential site for systemic *Salmonella* dissemination is the bone marrow, and previous investigations demonstrate *Salmonella* recovery from chicken bone marrow at a low prevalence rate, especially from flocks with a positive pathogen status^16,17^. Kassem et al. demonstrate efficient dissemination of *S*. Enteritidis to the bone marrow (100% prevalence) within 3 days of inoculating 1-day-old chicks with a pathogen dose of 2 × 10^5^ CFUs, and *S*. Enteritidis persisted in the bone marrow at 34 days post-inoculation in 20% of the chickens^18^. Several studies suggest that pathogen dissemination to the bone marrow may be a risk for *Salmonella* contamination of MSC^19,20^. Our data demonstrated that vaccination with BBS 1134 mitigated DSC *S*. Enteritidis dissemination to both the spleen and the bone marrow, supporting vaccination as an intervention strategy to reduce the risk associated with pathogen colonization of systemic sites and potential contamination during poultry product processing.

### *S*. Typhimurium vaccine strain BBS 1134 is a DIVA while inducing significantly higher levels of circulating IgY (IgG) in chickens

Previous research investigations in swine demonstrated that ELISA could be used to differentiate infected from vaccinated animals (DIVA) following immunization with the *S*. Typhimurium vaccine strain BBS 866^10,11^. To determine whether BBS 1134 is likewise a DIVA in chickens, ELISA was performed using pooled serum from mock- or BBS 1134-vaccinated chickens at 5 weeks of age (0 dpi) with the IDEXX *Salmonella* Enteritidis Antibody Test. Antibodies to *Salmonella* Enteritidis were not detected (data not shown), indicating that the BBS 1134 vaccine strain is also a DIVA in chickens, and vaccination with two vaccine doses does not interfere with the monitoring of flocks to determine exposure to *Salmonella* Enteritidis on the farm.

**Fig. 3:**
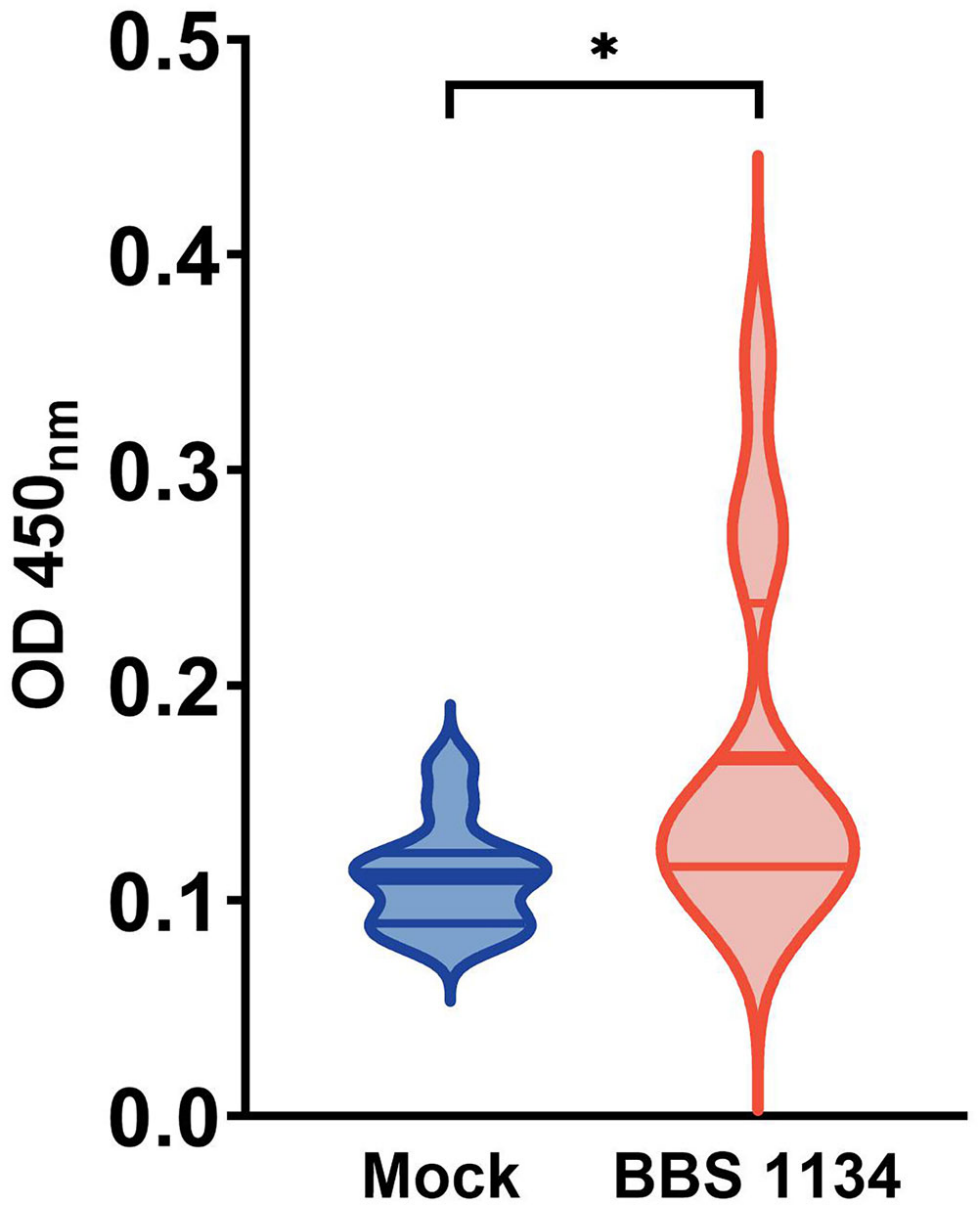
BBS 1134-vaccinated chickens have significantly higher levels of circulating IgY against the *Salmonella* vaccine strain. Serum samples were obtained from mock-vaccinated and BBS 1134-vaccinated chickens at 5-weeks of age (0 dpi; prior to DSC *S*. Enteritidis inoculation) and analyzed in an indirect ELISA for detection of IgY antibodies against the cell lysate of the BBS 1134 vaccine strain. The optical density (O.D._450_) of chromogen development is displayed on the Y-axis. Each plot represents a frequency distribution of the data. The thick line represents the mean and the thin lines indicate the quartiles. Vaccination procedures were performed as described in Fig. 1 legend. Statistically significant differences between groups are represented by *P ≤ 0.05.

Prior to DSC *S*. Enteritidis inoculation (0 dpi), serum was collected from the 5-week-old mock-vaccinated and BBS 1134-vaccinated chickens for indirect ELISA detection of IgY antibodies against the BBS 1134 vaccine strain. Although the individual chickens exhibited variability at low levels in each treatment group, a significantly higher level of IgY (*p* = 0.0283) against the vaccine strain was detected in the vaccinated chickens compared to the mock-vaccinated chickens (Fig. 3), indicating immune induction in the chickens vaccinated with the BBS 1134 vaccine strain.

### Microbiome diversity of mock-vaccinated and BBS 1134-vaccinated chickens prior to and following inoculation with DSC *S*. Enteritidis

**Fig. 4:**
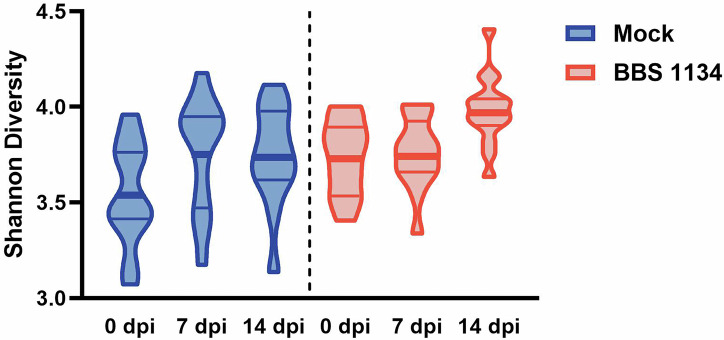
Shannon diversity index for mock-vaccinated and BBS 1134-vaccinated chickens at 0, 7, and 14 dpi. Cecal contents were obtained at each dpi and used for bacterial 16S rRNA gene sequencing and assessment of alpha diversity. Each plot represents a frequency distribution of the data. The thick line represents the mean and the thin lines indicate the quartiles.

To determine if vaccination altered the microbial community structure of the cecum, microbiome analysis was conducted on chicken cecal content samples via bacterial 16S rRNA gene sequencing at 0 dpi and following DSC *S*. Enteritidis SX514 inoculation (7 and 14 dpi). The resulting 532 amplicon sequence variants (ASVs) were evaluated for alpha diversity, a measure of richness and diversity within a sample. Shannon diversity of the main dataset significantly correlated with Observed ASVs (*S* = 44261, *p* = 5.5e-05, rho = 0.44), Fisher (*S* = 31012, *p* = 3.6e-09, rho = 0.61), and Simpson (*S* = 4718, *p* < 2.2e-16, rho = 0.94) diversities via Spearman rank correlation tests (Supplementary Data 1); thus, Shannon diversity was selected for alpha diversity evaluation. Collectively across groups, Shannon diversity was significantly different via Kruskal-Wallis analysis (*χ*^2^ = 19.03, *p* = 0.0019); however, pairwise Shannon diversity of the cecal microbiome was not significantly different (Benjamini-Hochberg adjusted (adj.) *p* > 0.05) either within a treatment group over time or across treatment groups at any specific timepoint (Supplementary Data 2; Fig. 4). While the literature has reported variable changes in the alpha diversity of the chicken gut microbiome in response to different combinations of vaccination and *Salmonella* challenge, the variations have generally not been statistically different^21–24^. Consistent with other studies^25,26^, a gradual (non-significant) increase of alpha diversity occurred as the richness and complexity of the microbiome increased with age for both treatment groups.

**Fig. 5:**
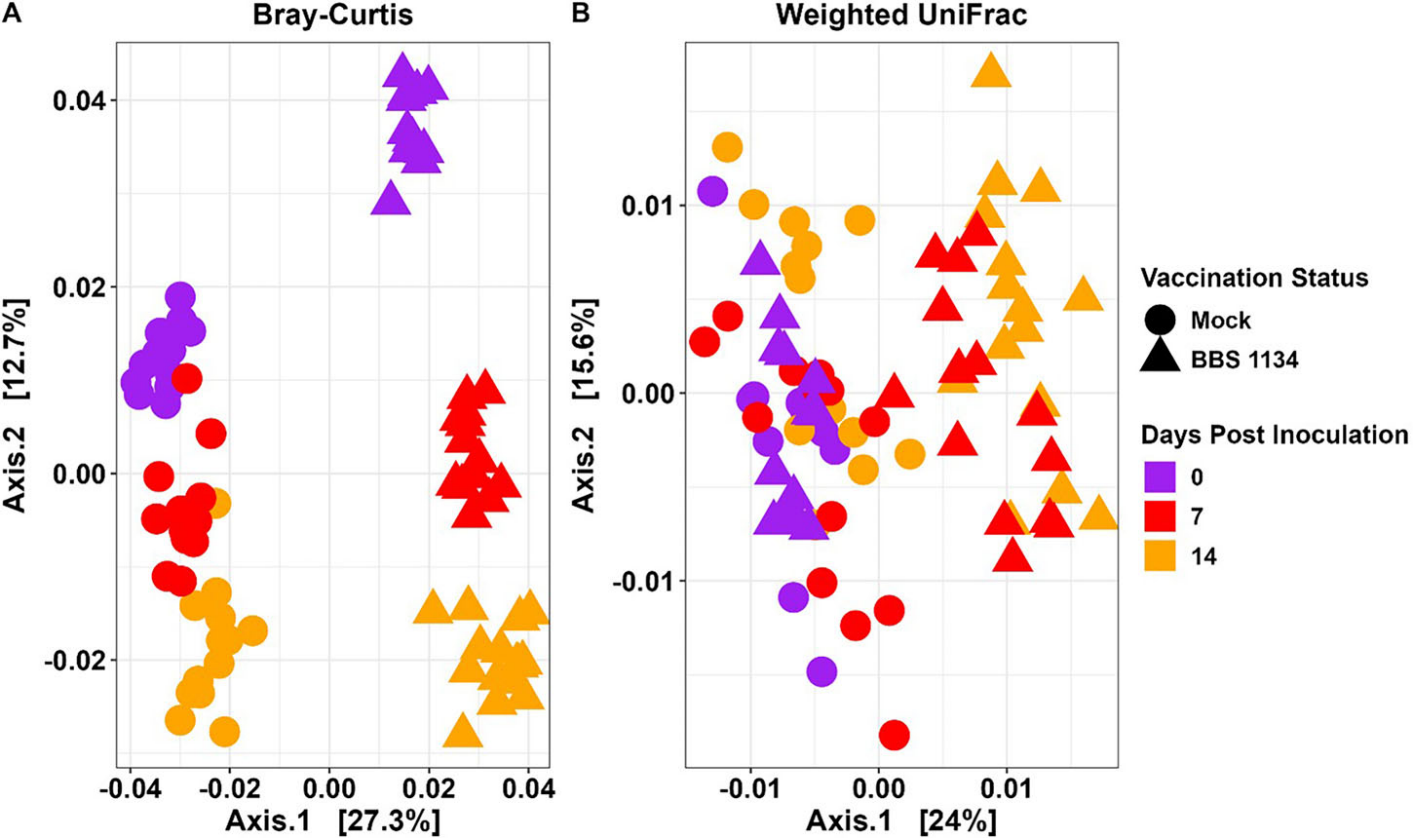
Beta diversity for mock-vaccinated and BBS 1134-vaccinated chickens at 0, 7, and 14 dpi. Bray-Curtis (**A**) and weighted UniFrac (**B**) distance matrices used as ordination inputs for principal coordinates of analysis. Axes 1 and 2 are shown with shape and color representing vaccination group and days post inoculation, respectively.

Bray-Curtis analysis of beta diversity (the difference in microbial composition between groups) revealed that prior to *S*. Enteritidis inoculation (0 dpi), the BBS 1134-vaccinated chickens had a distinct microbiome compared to the mock-vaccinated group (PERMANOVA *R*^2^ = 0.54, adj. *p* = 0.0001; Supplementary Data 3). Following SX 514 inoculation, microbiomes continued to be distinguishable between the two treatment groups through 14 dpi (Supplementary Data 3; Fig. 5A; 7 dpi *R*^2^ = 0.38, adj. *p* = 0.0001; 14 dpi *R*^2^ = 0.36, adj. *p* = 0.0001). A distinct clustering of mock-vaccinated versus BBS 1134-vaccinated samples was observed across principal coordinates analysis (PCoA) Axis 1 (27.3% of total variation) and within group separation based on time across Axis 2 (12.7%). While the Bray-Curtis index measures the compositional dissimilarity between microbial communities, weighted UniFrac considers the phylogenetic distances between community members weighted by relative abundance of their sequences^27^. Accounting for phylogeny with weighted UniFrac analysis, a visual separation between treatment groups at 7 and 14 dpi was observed across Axis 1 (24%) (Fig. 5B), but clustering was less distinct between 0 dpi samples and across time points than the Bray-Curtis plot (Fig. 5A). However, rotation of the axes for the weighted UniFrac plot revealed greater separation of clusters for mock-vaccinated and BBS 1134-vaccinated samples at 0 dpi across Axis 3 (13.2%) (Supplementary Fig. 1B). Similar to Bray-Curtis analysis, pairwise PERMANOVA analysis between vaccination statuses by dpi groups (*n* = 6) were significantly different based on weighted UniFrac pairwise analyses (adj. *p* < 0.05; Supplementary Data 3). These observations correspond with the literature, which generally shows significant beta diversity differences between vaccination and *Salmonella* challenge combinations^21–24^.

Reflecting a greater proportion of variability explained by time in the vaccinated group, PERMANOVA *R*^2^ values were higher across time within the BBS 1134-vaccinated groups (0 dpi vs 14 dpi = 0.42) than in mock-vaccinated groups (0 dpi vs 14 dpi = 0.34) for Bray-Curtis analysis (Supplementary Data 3). This pattern was also present in weighted UniFrac analysis where BBS 1134-vaccinated groups had higher *R*^2^ across time (0 dpi vs 14 dpi = 0.42) than mock-vaccinated groups (0 dpi vs 14 dpi = 0.20). Thus, the beta diversity analysis indicated that the mock-vaccinated group had less separation between 0 dpi and 14 dpi compared to the BBS 1134-vaccinated group, suggesting a greater change in the microbiome in the vaccinated chickens following *S*. Enteritidis challenge.

**Fig. 6:**
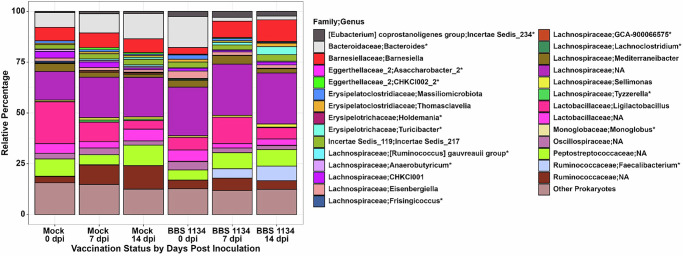
Relative abundance of bacterial genera for mock-vaccinated and BBS 1134-vaccinated samples at 0, 7, and 14 dpi. Genera were included if they had a mean relative percentage greater than 1% across all samples (Supplementary Data 5). ^*^Indicates the named genera identified by ANCOMBC2 as having statistically different abundances between vaccination types across each time point. Genera were considered significantly differentially abundant if they had *q*-values less than 0.5 and passed the ANCOMC2 sensitivity test (Supplementary Data 4). At 0 dpi, three genera (CHKCI002_2 (Eggerthellaceae_2 family), *Lachnoclostridium*, and Insertia Sedis_234 ([Eubacterium] coprostanoligenes group family)) were significantly higher in abundance and four genera (*Faecalibacterium*, *Asaccharobacter*_2, *Anaerobutyricum*, and *Frisingicoccus*) were significantly lower in abundance in the BBS 1134-vaccinated samples compared to mock-vaccinated samples (Supplementary Data 4). At 7 dpi, *Bacteroides*, GCA-900066575 (Lachnospiraceae family), and *Tyzzerella* were significantly higher in mock-vaccinated samples, while *Faecalibacterium* and *Turicibacter* were higher in BBS 1134-vaccinated samples. At 14 dpi, *Holdemania*, *Bacteroides*, and the [Ruminococcus] gauvreuii group were significantly higher in mock-vaccinated samples, while *Faecalibacterium* and *Monoglobus* were higher in BBS 1134-vaccinated samples.

Recent *Salmonella* vaccine investigations in chickens demonstrated that vaccination impacts the commensal cecal microbiota and that vaccine-mediated microbial community alterations may participate in the reduction of wildtype *Salmonella* colonization^21,22,24,28^. To explore microbial differences in the current study, bacterial genera were tested for differential relative abundance between mock-vaccinated and BBS 1134-vaccinated groups at each time point by ANCOMBC2 analysis (Fig. 6; Supplementary Data 4). *Tyzzerella* (7 dpi) and *Bacteroides* (7 and 14 dpi) in the mock-vaccinated group and Incertia Sedis_234 (0 dpi), *Faecalibacterium* (7 and 14 dpi), and *Turicibacter* (7 dpi) in the BBS 1134-vaccinated group were the only differentially abundant genera with relative percentages greater than 1% of the microbiome at a given time point (Supplementary Data 4 and 5). *Tyzzerella*, *Bacteroides*, *Turcibacter*, and *Faecalibacterium* have all been previously identified as part of the gut microbiota in chickens. Studies have demonstrated these genera have been associated with beneficial impacts on chicken host physiology such as: increased growth performance (*Faecalibacterium*), playing a role in bile acid, intestinal motility, and neurotransmitter regulation (*Turicibacter*), and either being producers of short-chain fatty acids (SCFA) or associated with stimulation of SCFA production (all)^21,29–33^. SCFAs include metabolites such as butyrate and can serve as a carbon source for enterocytes and enhance intestinal barrier function. Further, *Salmonella* abundance in the cecum, a preferred site of colonization, has been demonstrated to have a negative correlation with SCFAs. The post-DSC *S*. Enteritidis challenge microbiome data suggest functional redundancy with distinct microbial differences as some SCFA producing or stimulating genera were significantly higher in the mock-vaccinated group (*Bacteroides* and *Tyzzerella*), while other genera were significantly greater in the BBS 1134-vaccinated group (*Faecalibacterium* and *Turicibacter*).

In conclusion, *Salmonella* Enteritidis is a common serovar associated with chickens, and the emergence of isolates with decreased susceptibility to ciprofloxacin is a concern for public health. Pathogen interventions that can be applied during poultry production to limit *S*. Enteritidis colonization of chickens are needed to reduce contamination of the production environment and the food supply chain. Vaccination of broiler chickens with BBS 1134 significantly reduced DSC *S*. Enteritidis colonization of the cecum and systemic dissemination to the spleen. Furthermore, vaccination prevented dissemination of DSC *S*. Enteritidis to the bone marrow, a possible critical control point for product contamination during mechanical separation of meat from the bone. Similar to other investigations in swine, this study demonstrated that the BBS 1134 vaccine is a DIVA in chickens that will not interfere with flock level surveillance for natural *Salmonella* exposure on the farm. The cecal microbiome of BBS 1134-vaccinated chickens was distinct compared to mock-vaccinated birds, and potentially beneficial commensal bacteria associated with intestinal health were identified in the vaccinated chickens. Overall, the *Salmonella* BBS 1134 vaccine induced a cross-protective immune response against emergent DSC *S*. Enteritidis that reduced tissue colonization while maintaining DIVA status in broiler chickens and enhanced beneficial intestinal bacteria that may antagonize pathogen colonization.

## Methods

### Animal trial and bacteriology

Animal use and procedures followed humane protocols as approved by the USDA, ARS, National Animal Disease Center Animal Care and Use Committee in strict accordance with the recommendations in the Guide for the Care and Use of Laboratory Animals by the National Research Council of the National Academies. One day old, unsexed Cobb broiler chicks were obtained from a commercial hatchery and randomly distributed into two ABSL-2 isolation rooms (*n* = 44/room) at the National Animal Disease Center. Upon arrival, the intestines and yolks of 10 randomly selected chicks tested negative for *Salmonella* using previously described methods of qualitative bacteriology^34^. Chicks were fed ad libitum with Purina Game Bird Startena (Purina Animal Nutrition, Arden Hills, MN) from arrival to 3 weeks of age and then switched to Purina Flock Raiser for the remainder of the study. Simulating vaccination procedures in poultry production, chicks in one isolation room were individually administered ~3 × 10^8^ CFU (colony forming units)/chick of BBS 1134 vaccine (kanamycin-sensitive derivative of BBS 866)^12^ in phosphate buffered saline (PBS; Sigma-Aldrich, St. Louis, MO) via aerosolization on arrival and booster vaccinated via communal water administration at 2 weeks of age^35^. Chicks in the other isolation room, serving as the control, were mock vaccinated through the same methods with sterile PBS. At 5 weeks of age, all chickens were inoculated via oral gavage with 1 mL of PBS containing 1 × 10^9^ CFU of *S*. Enteritidis strain SX514 (FSIS12211648) with decreased susceptibility to ciprofloxacin due to a *gyrA* mutation encoding an amino acid substitution (D87Y).

At 0 (*n* = 12/12), 7 (*n* = 13/14), and 14 (*n* = 13/16) days post-inoculation (dpi) with DSC *S*. Enteritidis, chickens were randomly selected for tissue harvest and evaluation from both mock- and BBS 1134-vaccinated rooms following euthanasia with intravenous barbiturates per label dose. Terminal blood collections were performed at 0 dpi under intramuscular-injected anesthesia using serum separation tubes (BD Vacutainer, Becton, Dickinson and Co., Franklin Lakes, NJ); after clotting, serum was separated by centrifugation (1000 × *g*, 15 min, 4 °C) and stored at −80 °C. Bone marrow extraction was performed as previously described^35^ with modifications: sterile wooden applicators were used to remove the bone marrow from the medullary cavity. Extracted bone marrow was weighed and resuspended to 1 g/2 mL PBS. The cecum and spleen were aseptically collected from each chicken during tissue harvest, and were processed for qualitative and quantitative detection of *Salmonella* as previously described^34^. Cecal contents were collected for microbiome analysis and stored at −80 °C. XLT-4 medium (Becton, Dickinson and Co.) was supplemented with 50% Tergitol and 30 μg/mL nalidixic acid (Sigma-Aldrich). The identity of presumptive *Salmonella* colonies was confirmed by mauve colonies on BBL^TM^ CHROMagar^TM^ *Salmonella* (Becton, Dickinson and Co.) medium. Statistical analyses of cecum, spleen, and bone marrow quantitation were performed using Mann-Whitney tests in GraphPad Prism 10.4.0 (GraphPad Software, La Jolla, CA).

### IDEXX SE ELISA

The IDEXX *Salmonella* Enteritidis Antibody Test (IDEXX SE Ab X2 Test, Westbrook, ME) was performed as directed by the manufacturer using a 1:500 dilution of the chicken serum collected from mock-vaccinated and BBS 1134-vaccinated chickens (*n* = 12/group) at 5 weeks of age (prior to SX514 challenge).

### Indirect ELISA for serum IgY (IgG) detection

*Salmonella enterica* serovar Typhimurium strain BBS 1134 (vaccine) was streaked on Luria-Bertani (LB) agar and incubated at 37 °C. A single colony was inoculated into 3 mL LB broth and grown shaking overnight at 37 °C, then sub-cultured into 50 mL LB (1:1000 dilution) and incubated statically overnight. Cells were harvested by centrifugation at 3000 × *g* for 8 min, washed twice with an equal volume of sterile distilled water, and resuspended in 10 mL PBS. The bacterial suspension was heat-inactivated at 65 °C for 45 min with intermittent mixing; the whole cell lysate was confirmed for cell death by plating on LB and XLT4 agar, and stored in aliquots at −20 °C. Total protein concentration of the whole cell lysate was determined using the Pierce™ BCA Protein Assay Kit (Thermo Scientific, Rockford, IL), yielding a concentration of 287.2 µg/mL.

Immuno Maxisorp 96-well ELISA plates (Thermo Scientific) were coated with 200 µL/well of heat-inactivated BBS 1134 (1 µg/mL in carbonate (J.T.Baker, Radnor, PA)-bicarbonate (Sigma-Aldrich) coating buffer, pH 9.6) and incubated at 4 °C overnight. All subsequent incubations were performed at room temperature. Wells were washed (thrice with 300 µL of PBS containing 0.1% Tween-20 (Sigma-Aldrich) (PBS-T)) and blocked for 1 h with PBS containing 1% bovine serum albumin (BSA) (Sigma-Aldrich). Following another wash, individual serum samples from broiler chickens were diluted 1:800 in PBS-T and added to duplicate wells. Plates were incubated for 1 h, washed, and incubated with HRP-conjugated goat anti-chicken IgG (1:40,000 in PBS-T) (Bethyl, Boston, MA) for 1 h. After washing, 100 µL/well of TMB stabilized chromogen (Thermo Scientific) was added and incubated for 30 min in the dark. The reaction was stopped with 100 µL/well of 1 N sulfuric acid (Honeywell/Fluka, Muskegon, MI), and absorbance was measured at 450 nm using a BioTek Synergy HT plate reader (GEN5 software v2.5, Winooski, VT). Replicates for each animal were averaged before statistical analysis and datasets were tested for normality. Significant differences between the BBS 1134-vaccinated and mock-vaccinated groups were analyzed with GraphPad Prism 10.4.0 using a Mann-Whitney test.

### Microbiome analysis

Total microbial DNA was extracted from chicken cecal content samples using the QIAamp PowerFecal® Pro DNA Kit (Qiagen, Germantown, MD). Extractions were performed in batches of 12 with approximately two of each treatment-timepoint group, with some variation due to differences in group size. DNA was assessed qualitatively by agarose gel electrophoresis and quantitatively using the Qubit^TM^ dsDNA Broad Range Quantitation Kit (Invitrogen, Waltham, MA). DNA samples were standardized to 10 ng/µL in UltraPure distilled water, and PCR amplification of the V4 region of 16S rRNA gene was performed as previously described^36,37^. Sterile, nuclease-free water was used as a negative control, and a mock community consisting of an even mix of the genomic material for 20 known bacterial strains (ATCC MSA-1002; ATCC, Manassas, VA) was used as a positive control. Successful amplification was confirmed by agarose gel electrophoresis and Qubit^TM^ dsDNA High Sensitivity Quantitation Kit (Invitrogen). Individual samples were normalized using SequelPrep^TM^ Normalization Plates (Applied Biosystems, Waltham, MA), pooled, and concentrated using the Zymo Research DNA Clean and Concentrator® (Zymo Research, Irvine, CA). All samples were sequenced using the Illumina MiSeq platform (Illumina, San Diego, CA) with a V2 reagent kit (2 × 250 bp read lengths).

Amplicon sequence variants (ASVs) were delineated with DADA2 (*n* = 2066 total ASVs) and filtered to remove ASVs not annotated at the Kingdom level (*n* = 40), annotated as Archaea (*n* = 1), and considered to be contaminants by the decontam R package (v. 1.24.0) frequency-based method (*n* = 5)^38–41^. The negative (*n* = 1) and positive (*n* = 1) controls were removed as well as samples with less than 10,000 sequences (*n* = 1; bird 12 from BBS 1134-vaccinated group at 0 dpi (V514_D0_12)), resulting in removal of 41 ASVs exclusive to those samples (Supplementary Data 6). Alpha and beta diversity analysis was performed on the resulting dataset in R using the phyloseq R package (v. 1.48.0)^42^. Spearman rank correlation tests were used to test correlations between alpha diversity metrics (Shannon, Simpson, Fischer’s, Observed ASVs), while Kruskal-Wallis with Dunn pairwise tests were performed for statistical significance between metadata groups for Shannon diversity. Beta diversity between samples was calculated using variance-stabilizing transformed counts (DESeq2 v. 1.44.0) with Bray-Curtis and weighted UniFrac, and statistical differences between metadata groups, overall and pairwise, were calculated with permutational analysis of variance (PERMANOVA)^43^. ASVs with less than 10 sequences were filtered to remove low abundance ASVs (*n* = 185) prior to detecting differentially abundant genera with the ANCOMBC2 (Analysis of Compositions of Microbiomes with Bias Correction 2) R package (v. 2.6.0)^44,45^ (Supplementary Data 6 and 7). Genera were considered significantly differentially abundant if they had *q*-values less than 0.5 and passed the ANCOMC2 sensitivity test. Figures were created with ggplot2 R package (v. 3.5.2)^46^.

## Supplementary information

Supplementary Figure 1 011226

Bearson et al Chicken Vaccine Enteritidis 16S_cecal Supplementary Data 011426

## Data Availability

Raw 16S reads were uploaded to the NCBI Sequence Read Archive under BioProject PRJNA1309157.

## Code availability

R scripts and full R session information with loaded libraries and their versions are located on GitHub (https://github.com/USDA-FSEPRU/bbs_1134_vaccine_vs_dsc_enteritidis_challenge.git).
