# Supplementary material for: Chicken vaccination reduces colonization and dissemination of Salmonella serovar Enteritidis with decreased susceptibility to ciprofloxacin
Source: NPJ Vaccines. 2026 Mar 9;11:88. doi: 10.1038/s41541-026-01414-y (PMC13100210; doi:10.1038/s41541-026-01414-y)
Supplement: Supplementary file 1 — Supplementary Figure 1 011226 [file 41541_2026_1414_MOESM1_ESM.pdf]

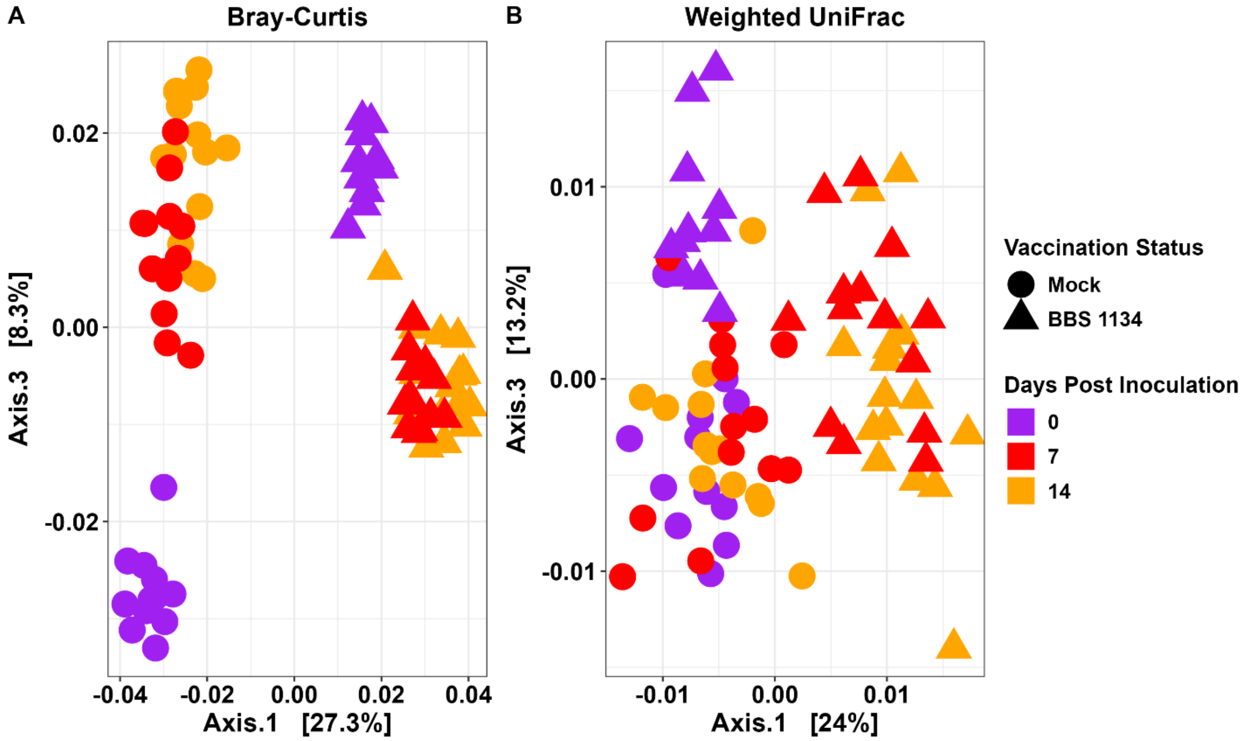

Supplementary Figure 1. Beta diversity for mock- and BBS 1134-vaccinated chickens for 0, 7, and 14 days post-inoculation (dpi). Bray-Curtis (A) and weighted UniFrac (B) distance matrices used as ordination inputs for principal coordinates of analysis. Axes 1 and 3 are shown with shape and color representing vaccination group and days post inoculation, respectively.
